# Effect on Satiety-Related Biomarkers of Bar Snacks Containing Chickpea Flour and Pork Protein

**DOI:** 10.3390/nu16183180

**Published:** 2024-09-20

**Authors:** María-Dolores Zomeño, Mireia Malcampo, Karla Alejandra Pérez-Vega, Antoni Pastor, Maria López-Roura, Begoña Arrufat, Sergio Atarés, Sergio José Ramos, David Alonso, Isaac Subirana, Daniel Muñoz-Aguayo, Gemma Blanchart, Sònia Gaixas, Marta Cabañero, Susanna Tello, Valentini Konstantinidou, Javier Hernando-Redondo, Albert Goday, Olga Castañer, Helmut Schröder, Montserrat Fitó

**Affiliations:** 1 Hospital del Mar Research Institute, 08003 Barcelona, Spain; mzomeno@researchmar.net (M.-D.Z.); mmalcampo@researchmar.net (M.M.); kperez@researchmar.net (K.A.P.-V.); apastor@researchmar.net (A.P.); marialopezroura@gmail.com (M.L.-R.); isubirana@researchmar.net (I.S.); dmunoz@researchmar.net (D.M.-A.); gblanchart@researchmar.net (G.B.); mcabanero@researchmar.net (M.C.); jhernando@researchmar.net (J.H.-R.); agoday@psmar.cat (A.G.); hschroder@researchmar.net (H.S.); mfito@researchmar.net (M.F.); 2 Consortium Center for Biomedical Research Network (CIBER), M.P. Pathophysiology of Obesity and Nutrition (CIBERobn), Instituto de Salud Carlos III, 28029 Madrid, Spain; stello@researchmar.net; 3 Blanquerna School of Health Sciences, Universitat Ramon Llull, 08025 Barcelona, Spain; 4 Fertinagro Biotech S.L., 44195 Teruel, Spain; bego.arrufat@tervalis.com (B.A.); sergio.atares@tervalis.com (S.A.); 5 Naturuel S.L., 44002 Teruel, Spain; sergio.ramos@naturuel.com (S.J.R.); david.alonso@naturuel.com (D.A.); 6 Consortium Center for Biomedical Research Network (CIBER), M.P. Cardiovascular Diseases (CIBERcv), Instituto de Salud Carlos III, 28029 Madrid, Spain; 7 Consortium Center for Biomedical Research Network (CIBER), M.P. Epidemiology and Public Health (CIBEResp), Carlos III Health Institute, 28029 Madrid, Spain; 8 DNANUTRICOACH®—Nutrigenetics Center, 16674 Athens, Greece; vkonstantinidou@gmail.com; 9 Department of Medicine, Autonomous University of Barcelona, 08193 Barcelona, Spain

**Keywords:** satiety, plant protein, endocannabinoid compounds

## Abstract

This project aims to establish the acceptability and satiety of a hybrid snack containing plant protein and a small percentage of animal protein compared to a meat-based snack. Design: Randomised, crossover, double-blind, controlled post-prandial trial involving 24 participants (18–30 years), with two interventions: (a) a hybrid snack containing plant protein derived from chickpeas and 6.6% lean high-quality pork meat; and (b) a meat-based snack containing 90% lean pork meat. Methods: General, life-style, sensory acceptability questionnaire, and the following laboratory analyses were performed: lipid profile, endocannabinoids, and related compounds. Results: Sensory questionnaires showed in general good acceptability for both bars. Additionally, there was a greater increase in glycemia at 30, 60, and 90 min after consuming the hybrid snack compared to the meat-based snack, with no changes in the lipid profile. Regarding the endocannabinoid compounds and related compounds, the compound N-palmitoleoyl ethanolamine in the acylethanolamide group showed higher levels overall following the consumption of the hybrid snack compared to the meat-based snack, particularly at 2 h. Conclusions: The hybrid snack was associated with changes in endocannabinoid-like compounds. Therefore, it may provide a lasting satiating effect, while complementing the protein profile of plant-based foods with the quality of animal protein.

## 1. Introduction

Meat and meat products constitute a relevant part of the dietary pattern in Spain and Europe in general. Meat is composed of water, proteins, fats and fatty acids, vitamins, and other bioactive components such as minerals. In this sense, meat represents a source of essential aminoacids for the growth and development of the organism. From a nutritional standpoint, meat intake involves acquiring high-quality proteins, with animal protein being of high biological value as it contains all the amino acids (essential and non-essential). A total of 36% of global meat consumption is pork, 35% is poultry, and 22% is beef [1]. Regarding the pork used in sausage production, it is worth noting its nutritional presence of complete proteins, vitamin B12, Zn, and heme iron (more easily absorbed than non-heme iron, and does not require vitamin C to enhance its bioavailability) [2,3,4].

The meat industry is currently experiencing a period of great innovation to differentiate its products and meet the quality and sustainability expectations of consumers. Thus, the innovation process extends to all their products, including sausages or other processed meats made from minced meat. According to the Spanish legislation (BOE: the primary source for disseminating legal and administrative and information in Spain), non-heat-treated meat derivatives, including sausages, are defined as products made with meat or meat and fat that have not undergone any treatment or have undergone a curing–ripening process, with or without fermentation, drying, marinating, or other non-thermal technological processes sufficient to confer their organoleptic characteristics. These products may contain cereals or other plant-based ingredients in their composition. One of the ingredients that can be used in the production of sausages is chickpea flour, which is obtained by grinding dried chickpeas [4].

Legume seeds, widely consumed in Mediterranean countries, include foods such as chickpeas, beans, lentils, soybeans, broad beans, and peas, among others. This group of foods stands out for their high nutritional value, as well as their economic cost and easy storage. Their main macronutrient is complex carbohydrates, which are beneficial due to their slow absorption. Additionally, they are rich in plant-based proteins (19–36%), particularly the amino acids lysine and arginine, in contrast to proteins rich in sulphur-containing amino acids (methionine and cysteine), which are predominant in cereals. Furthermore, they are significant sources of vitamins from the B group (B3, B6, and B9) and minerals (Ca, P, Mg, Fe, Zn, and K). Regarding iron, chickpeas have the highest bioavailability compared to other legumes (91%), and it is in the non-heme form. This is why it is recommended to consume chickpeas together with foods rich in vitamin C to enhance its absorption. In the case that whole chickpeas, including the skin, are used in the production of chickpea flour, its dietary fibre content, which facilitates intestinal transit, regulates carbohydrate absorption, and positively affects the composition of the intestinal microbiota. Furthermore, legumes have a wide variety of phenolic compounds (condensed tannins, phytoestrogens) [2,3]. Regarding the functional properties of chickpea flour, its remarkable water absorption capacity influences the texture of meat products in which it is used, providing consistency and viscosity [5].

The three macronutrients have varying effects on satiety, with protein being the most satiating and fat the least. With regard to their thermic effect, protein induces the highest and fat the lowest thermic response following isocaloric ingestion [6].

Our aim is to analyse the acceptability and satiety of a hybrid snack containing plant-based protein from chickpeas and 6.6% high-quality pork meat, with the objective of complementing and improving the quality of the consumed protein compared to the consumption of a high-quality pork sausage-snack.

## 2. Materials and Methods

### 2.1. Study Design

A randomised, crossover, and controlled clinical post-prandial trial was conducted on 24 healthy individuals to evaluate the post-prandial effect of consuming snacks rich in plant and, to a lesser extent, animal protein compared to a high-quality pork sausage snack. Each participant will consume both types of snacks (20 g) in a randomly assigned order.

During the initial interaction with potential participants, the study, its objectives, procedures, confidentiality, and the requirement to sign an informed consent were explained. The study was conducted according to a protocol reviewed by the Ethics Committee of Hospital del Mar (Protocol Code: Fertinagro IDI-20190984). In addition, the study was registered at ClinicalTrials.gov (ID: NCT05375175). The CONSORT checklist is described in Supplementary Table S1.

### 2.2. Study Population

An informative infographic was used to recruit participants from frequented locations for the targeted age range, such as universities and our own research center, Biomedical Research Park of Barcelona (PRBB). Individuals who expressed interest in the study underwent screening for exclusion criteria. Those who met the criteria were provided with an explanation of the study and scheduled for an interview to discuss the study in detail and obtain their informed consent.

Inclusion Criteria: a total of 24 participants aged between 18 and 30 years were included in the study, with a gender ratio of 50% men and 50% women.

Exclusion criteria: Diabetic condition, chronic medication use except contraceptives, diagnosis of inflammatory bowel disease, any severe active disease that impedes or incapacitates the ability to properly follow the study, regular cannabis consumption, active alcoholism or drug dependency, inability to provide informed consent, pregnancy, and intolerance to any of the components of the snacks.

### 2.3. Intervention Foods

Participants perform two post-prandial interventions with both snacks (see nutritional composition in Table 1).

(a) Dehydrated baked snack primarily composed of vegetal protein with a small portion of meat (6.6%). Physicochemical characteristics: moisture content 6.62%, water activity (AW) 0.492, and pH 5.5.

(b) Dehydrated snack, primarily composed of meat (90%), was prepared through baking. Physical-chemical characteristics: moisture content of 20.02%, water activity (AW) of 0.751, and a pH of 6.02.

Both snacks were packaged in 20 g bags made of metallized PET/PE laminate. They were packed under a controlled atmosphere of N2 with oxygen absorbers to extend their shelf life. The products were sealed and labelled with a batch number, and they were stored in a cool, dry place without refrigeration throughout their shelf life.

### 2.4. Post-Prandial Intervention

The randomization was conducted using blocks of 5 participants and implemented with R language software (version 4.3.3). Totals of 11 and 13 participants were included in the two randomization order groups for starting with the hybrid snack or meat-based one. To ensure double-blinding, the packaging of the snack bars was identical, and the nursing staff and the statistical technician could only distinguish the products by their assigned codes. The intervention involved administering 20 g of either snack, which was consumed in a fasting state in the early morning within a 6 min period. Afterwards, participants were given 150mL of water to rinse their mouth and ensure complete consumption of the snack bar. Participants arrived after a 12 h fasting period, avoiding solid foods or liquids, and more than 1 h after water intake, in the early morning (7:45 a.m.). They were placed in a resting state on a stretcher with an intravenous line for the collection of blood samples prior to snack ingestion. Vital signs were measured. To study the post-prandial response to the snack ingestion, blood samples were collected at baseline and 15 min, 30 min, 60 min, 90 min, and 120 min (6 time points). Following a 20-day interval, the intervention was repeated with the second type of snack according to randomization, following the same process. The total duration of the study for participants was approximately 2.5–3 months.

No dietary or lifestyle interventions (such as diet, physical activity, etc.) were conducted in the clinical trial. Any incidents were referred to the participants’ primary care physician.

### 2.5. Sensorial Properties

A sensory test was administered to assess the acceptability of the administered food product. A rating from 1 to 5 was requested for the preference in colour, flavour, and texture, with a higher score indicating a greater acceptability of the product.

### 2.6. Study Variables

General data questionnaire was administered at the beginning of the study. A 14 item-score diet questionnaire for establishing adherence to the Mediterranean diet was administered at the beginning of each of the two interventions [7]. Finally, a physical activity questionnaire was administered at the beginning of each of the two interventions. This was carried out using a shortened version of the Minnesota Leisure Time Physical Activity Questionnaire [8].

### 2.7. Metabolic Variables

Analysis were conducted with samples at baseline before the intake of the snack and at 15 min, 30 min, 60 min, 90 min, and 120 min after consumption. Serum levels of glucose, total cholesterol, HDL cholesterol, and triglycerides were measured by standard enzymatic methodologies in a PENTRA-400 equipment (ABX Diagnostic, Montpellier, France). LDL cholesterol was calculated by the Friedwald formula whenever triglycerides were inferior to 300 mg/dL. In addition, insulin, ghrelin, and leptin were simultaneously analysed in serum by Bio-Plex Pro methodology, a bead-based multiplexing technology with specific capture antibodies coupled with magnetic beads to discriminate analytes using an XMAG-Luminex assay (Bio-Rad, Hercules, CA, USA). After several washes to remove unbound protein, a biotinylated detection antibody conjugated with fluorescent reporter dye was used. The fluorescence signal was read on a Bio-Plex 200 equipment (Bio-Rad) [9].

### 2.8. Endocannabinoids and Related Compounds

Analyses were conducted with samples obtained from the same six time points as the metabolic variables (see the paragraph above).

For endocannabinoid sample collection, EDTAK2 blood tubes (Polymouth, UK) were immediately centrifuged at 1700× *g* for 15 min at 4 °C after extraction. Prior to freezing, Orlistat solution was added to the collected plasma as a preservative for the analysis of endocannabinoids and related compounds following a previously validated method [10]. Briefly, aliquots of 0.5 mL of human plasma were transferred to 12 mL glass tubes, spiked with deuterated internal standards (N-arachidonoylethanolamine-d4, N-docosahexaenoylethanolamine-d4, N-linolenoyl ethanolamine-d4, N-oleoyl ethanolamine-d4, N-palmitoleoyl ethanolamine-d4, N-palmitoyl ethanolamine-d4, 2-arachidonoyl glycerol-d5 and 2-oleoyl glycerol-d5), diluted with 0.1 M ammonium acetate buffer (pH 4.0), and extracted with tert-butyl methyl ether. The dry organic extracts were reconstituted in 100 μL of a mixture water as follows: acetonitrile (10:90, *v*/*v*) with 0.1% formic acid (*v*/*v*) and transferred to HPLC vials. A Waters Acquity UPLC system with a Xevo TQ-Smicro Mass Spectrometry detector (Milford, MA, USA) was used for the analysis. Chromatographic separation was performed with a Waters BEH-C18 column (2.1 × 100 mm, 1.8 μm particle size) maintained at 40 °C with a mobile phase flow rate of 0.4 mL/min. The composition of the mobile phase is detailed as follows: A: 0.01% (*v*/*v*) formic acid in water and B: 0.01% (*v*/*v*) formic acid in acetonitrile. The mass spectrometry analysis was performed using the multiple reaction monitoring mode (MRM). Quantification was performed by isotope dilution. The deuterated internal standards were obtained from Cayman Chemical (Ann Arbor, MI, USA) and Toronto Research Chemicals (North York, ON, Canada), and solvents were from Merck (Darmstadt, Germany).

### 2.9. Sample Size Calculation

Accepting an alpha risk of 0.05 and a beta risk lower than 0.2 in a bilateral test, 23 subjects are needed to detect a difference equal to or greater than 0.08 units in endocannabinoid levels. A standard deviation of 0.135 is assumed. A loss-to-follow-up rate of 0% has been estimated [11].

### 2.10. Statistical Analysis

The results are represented as means +/- standard deviations. The normality of variables has been assessed through normality plots and skewness and kurtosis analysis. The log-transformation of variables was applied to achieve normality if necessary. A general linear model for repeated measures has been fitted. The interaction term between snack type and time has been assessed to evaluate the effect of snack on each variable. The time variable has been considered as a maximal degree polynomial, i.e., the number of times minus one. The analyses have been conducted on an intention-to-treat basis. Statistical software SPSS (version 22) and the open-source programming language R Software (version 4.3.3) have been used.

## 3. Results

A descriptive table of baseline results for the glucose and lipid profile, participants’ physical activity and adherence to the Mediterranean diet are shown in Table 2. The median and 1st and 3rd quartile of endocannabinoid compounds and related compounds are represented in Table 3.

### 3.1. Systemic Lipid Profile and Glycemia

As expected, a more pronounced and significant increase in serum glucose levels at 30 and 60 min after consuming the chickpea bar compared to the meat bar was observed, with the significance diminishing at 120 min.

### 3.2. Sensory Preferences

Regarding the sensory questionnaires, it was observed that the meat bar had a higher score in terms of colour, while the chickpea bar had a higher score in terms of texture, and there was a trend towards a higher score in taste (*p* = 0.052) (Table 4). Higher values towards the right/high corner means a better acceptability of the hybrid snack, whereas higher values towards the left-low corner means better acceptability of meat-based snack.

### 3.3. Endocannabinoid Compounds and Related Compounds

A battery of endocannabinoid compounds and related compounds (endocannabinoid-like compounds) has been analysed, including compounds from two groups: (a) N-acylethanolamines: N-arachidonoyl ethanolamine (anandamide, AEA), N-di-homo-γ-linolenoyl ethanolamine (DGLEA), N-docosahexaenoyl ethanolamine (DHEA), N-linolenoyl ethanolamine (LEA), N-oleoyl ethanolamine (OEA), N-palmitoleoyl ethanolamine (POEA), and N-palmitoyl ethanolamine (PEA); and (b) 2-monoacylglycerols: 2-arachidonoyl glycerol (AG), 2-linoleoyl glycerol (LG), and 2-oleoyl glycerol (OG).

No significant differences were observed between the baseline levels of the two randomization order groups. Significant changes were observed in the compound N-palmitoleoyl ethanolamine (POEA), particularly an increase at 2 h after consuming the hybrid snack compared to consuming the meat-based snack (Table 5).

**Figure 1:**
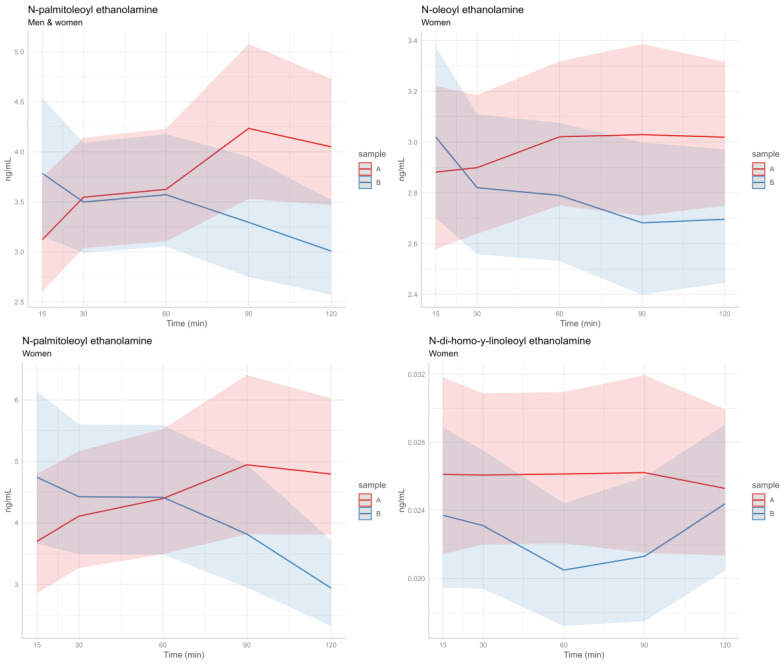
Kinetics of significant endocannabinoid-like compounds after consuming both snacks are depicted. A = hybrid snack; B = meat-based snack.

In Figure 1, the kinetics of both men and women (upper left quadrant) of the compound N-palmitoleoyl ethanolamine after consuming both snacks are depicted.

The analyses were conducted separately for men (n = 12) and women (n = 12). In men, the results are consistent with those described for the overall group of 24 subjects. In the case of women, a change at 120 min was observed in N-palmitoleoyl ethanolamine, similar to what was detected in the overall sample. Additionally, a change (with a trend towards significance) was observed at 120 min for N-oleoyl ethanolamine (OEA) and at 60 min for N-di-homo-y-linoleoyl ethanolamine (DGLEA) (Table 6).

In Figure 1, the kinetics in women of the compounds N-palmitoleoyl ethanolamine (left lower quadrant), N-oleoyl ethanolamine (right upper quadrant), and N-di-homo-y-linoleoyl ethanolamine (right lower quadrant) after the consumption of both snacks are depicted.

## 4. Discussion

In this randomised crossover trial, both bars, the hybrid and plant-based snack, were well-accepted by the participants. The satiating effect may last longer with the hybrid snack and, in general, was more pronounced in women.

Foods with a high content of complex carbohydrates and fibre have satiating effects. In the present study, specifically, a greater satiating effect was expected with the hybrid snack, given that the fibre content of chickpea flour is higher compared to that of dried chickpeas. Furthermore, chickpea flour is rich in soluble fibres; this type of fibre helps retain water and thus slows down digestion, which has positive effects on the gastrointestinal tract and cholesterol and glucose metabolism. It is known that the most satiating macronutrient is protein, and when combined with fibre, it promotes the incomplete absorption of certain nutrients, including fat [12]. This is why these hybrid foods could have higher satiety index than fat itself, thereby promoting adherence to the diet and maintaining body weight.

The present study indicates that significant changes occur in the group of endocannabinoid-like compounds known as acylethanolamides, specifically in the compound N-palmitoleoyl ethanolamine. The only two compounds with affinity for endocannabinoid receptors (CB1 and CB2) are anandamide (AEA) and 2-arachidonoyl glycerol (AG).

Endocannabinoid-like compounds, also known as ‘endocannabinoid-related’ compounds, are bioactive lipids that interact with the endocannabinoid system and related pathways. These compounds can affect neurotransmitter release in the brain areas involved in appetite regulation, such as the hypothalamus. Among others, they can modulate the release of dopamine and other neurotransmitters involved in reward and pleasure, thereby influencing food intake. Additionally, endocannabinoid and endocannabinoid-like compounds can modulate the release of gastrointestinal hormones like ghrelin, which stimulates appetite, and leptin, which promotes satiety. In summary, endocannabinoids play a role in the regulation of energy sensing related to hunger and satiety, as well as reward-driven motivation, thereby contributing to energy balance and the regulation of food intake [11]. Although an increase in anandamide levels was expected as it serves as a satiety marker, the results do not show significant changes. Nevertheless, all endocannabinoids and related compounds have bioactivities in the satiety/appetite axis [11]. In the present study, their levels are generally higher after consuming the hybrid snack compared to the meat-based snack, particularly around the 2 h mark. We specifically observed an increase in the POEA compound, 2 h after the hybrid snack intake in comparison with the meat-based snack. The compounds POEA and OEA act as potent agonists of the GPR119 receptor [13]. GPR119 is a lipid sensor indirectly involved in the secretion of incretin hormones by enteroendocrine cells of small intestine in response to food intake. This receptor’s role in the satiety/appetite axis and insulin metabolism through GLP1-1 release is still being established [14,15]. The activation of gastrointestinal GPR119 by microbiota-produced OEA derived from oleic acid was related to satiety control under energy-shortage conditions [16]. The fact that the specimen is plasma could explain why we have not observed an OEA increase, given that the OEA secretion has been described particularly in the small intestine [17]. Nevertheless, we could detect an increase in OEA, N-di-homo-y-linolenoyl ethanolamine (DGLEA)*,* and especially POEA when the analysis was performed in women. This factor could indicate that a higher dose than 20 g, which was employed in the present study, could be required in men to induce satiety.

The hybrid snack developed for the present study improves the nutritional profile of conventional sausages, since it is mostly composed of plant-based foods, containing only 6.6% high-quality lean pork to complete the protein profile. Furthermore, it is a product rich in slow-digesting carbohydrates and fibre. Due to its high content of chickpea flour, it exhibits characteristics that respect and promote sustainability, as legumes fix nitrogen in crop soil and are not required to be refrigerated, making transportation more economical. This hybrid product offer promotes local foods, and in addition, the innovation has produced higher-quality products that can meet consumer expectations in different areas.

It is important to highlight the role of satiety in controlling obesity from an early age, including in children and adolescents, and its significance in preventing obesity. The increasing prevalence of obesity in adolescents and children is a relevant public health problem in both developed and developing countries. To address obesity, there has been significant interest in understanding the relationship between different foods and nutrients and their effects on appetite regulation, body weight, and body composition. This supports the consumption of foods such as whole grains, legumes, and vegetables, which provide appropriate sources of carbohydrates and are associated with a reduced risk of cardiovascular and other chronic diseases, rather than foods high in sugars, such as sugar-sweetened beverages [18]. On the other hand, high-protein diets could be an alternative, given that protein confers a satiety effect that may favour energy intake control [19].

Plant-based diets require fewer resources such as land and water, and they generally have a lower carbon footprint compared to diets rich in animal products. On the other hand, the meat industry is a significant contributor to greenhouse gas emissions, deforestation, and water pollution. In this regard, young people especially are increasingly interested in environmentally sustainable diets, and reducing meat consumption is one of the first steps which can help mitigate these environmental impacts. In concordance, the industry is adapting to these societal demands [20]. To prevent global warming, the implementation of environmental, social, and governance (ESG) criteria is required [21]. Thus, food companies are moving towards investing in products that are both healthy and sustainable.

The first strength of our study is its randomised crossover design, which was conducted among free-living individuals. While the crossover design increases efficiency, a larger sample size would enhance the reliability and generalizability of the findings. A second strength is our comprehensive assessment of endocannabinoids and related compounds. However, as a limitation, our results, which are based on subjects aged 18–30 years, may not be extrapolated to other age groups, such as children or older adults. To address this, additional post-prandial, mid-, and long-term trials would be needed to confirm these findings. Furthermore, the results should be carefully interpreted in the context of the actual differences in nutritional composition within the present study design. Finally, a larger sample size would also be necessary to adequately examine potential sex differences.

## 5. Conclusions

Both the hybrid plant-based snack and the meat one were well-accepted by young participants. Regarding satiety, it was observed that the satiating effect may last longer with the hybrid snack, as there were differences at 2 h between the two interventions in N-palmitoleoyl ethanolamine, a compound associated with satiety. Therefore, the hybrid snack, which combines plant protein from chickpeas and 6.6% lean pork meat of high quality, provides a sustained satiety effect, mainly in women, while improving the protein profile of plant-based foods with the quality of animal protein.

The longer-lasting satiety effect of the hybrid snack compared to the meat snack could be attributed to (a) a higher amount of soluble fibre from chickpea flour compared to dry chickpeas; and (b) the protein composition, with a small proportion derived from meat.

## Figures and Tables

**Table 1:** Nutritional composition.

Nutritional Composition per 100 g	Hybrid Snack	Meat-Based Snack
Energy	1400–1600 kJ	1600–1900 kJ
	(200–400 kcal)	(300–500 kcal)
Total fat	5–20 g	25–40 g
Fatty acids		
Saturated	1–4 g	10–15 g
Monounsaturated	2–10 g	13–19 g
Polyunsaturated	2–6 g	2–6 g
Carbohydrates	30–45 g	1–6 g
Sugar	4–15 g	1–3 g
Protein	20–35 g	30–45 g
Fibre	10–25 g	1–3 g
Sodium	50–150 mg	1000–1500 mg
Others	- No added sugars	- No added sugars
	- Without artificial additives E	- Without artificial additives E
	- No allergens	- No allergens

**Table 2:** General and anthropometric characteristics of participants.

	Mean (SD)	n
Age (years)	23.6 (2.12)	24
Gender (% women)	12 (50.0%)	24
Height (cm)	171 (7.89)	24
Weight (kg)	63.4 (10.1)	24
Body Mass Index (kg/m^2^)	21.6 (2.52)	24
Systolic blood pressure (mm/Hg)	113 (8.42)	18
Diastolic blood pressure (mm/Hg)	64.6 (7.45)	18
Fasting Glucose (mg/dL)	82.2 (5.74)	24
Total cholesterol (mg/dL)	156 (31.7)	24
HDL cholesterol (mg/dL)	57.1 (14.1)	24
LDL cholesterol (mg/dL)	87.0 (24.9)	24
Triglycerides (mg/dL)	60.3 (28.0)	24
Insulin (pg/mL)	111 (95.3)	20
Ghrelin (pg/mL)	334 (165)	24
Leptin (pg/mL)	1990 (1784)	23
Physical activity (MET* min/week)	1907 (3165)	23
Mediterranean diet adherence (14 item-score)	9.11 (1.81)	18

HDL, high density lipoprotein; LDL, low density lipoprotein; SD, standard deviation, *MET, Metabolic Equivalent of Task.

**Table 3:** Median and 1st and 3rd quartile of endocannabinoid compounds and related compounds.

	Median (1st Quartile; 3rd Quartile)	n
og	2.31 (1.37; 3.01)	20
aea	0.21 (0.15; 0.23)	20
dglea	0.03 (0.02; 0.04)	20
dhea	0.23 (0.20; 0.32)	20
lea	0.58 (0.49; 0.70)	20
oea	3.22 (2.68; 3.67)	20
poea	5.36 (2.21; 10.0)	20
pea	6.53 (1.41; 8.65)	20
ag	0.60 (0.45; 0.90)	24
lg	1.36 (0.90; 1.67)	24
ratio_oea_aea	15.2 (13.1; 17.8)	20
ratio_oea_pea	0.46 (0.28; 2.87)	20
ratio_pea_aea	36.3 (6.66; 49.8)	20

og, 2-oleoyl glycerol; aea, N-arachidonoyl ethanolamine (anandamide); dglea, N-di-homo-y-linolenoyl ethanolamine; dhea, N-docosahexaenoyl ethanolamine; lea, N-linolenoyl ethanolamine; oea, N-oleoyl ethanolamine; poea, N-palmitoleoyl ethanolamine; pea, N-palmitoyl-ethanolamine; ag, 2-arachidonoyl glycerol; lg, 2-linoleoyl glycerol.

**Table 4:** Colour, flavour, and texture preferences.

Number of Participants According to the Score of the Hybrid Snack (Rows) and Meat-Based Snack (Columns)															
	Colour (*p* Value = 0.025 *)					Flavour (*p* Value = 0.052 *)					Texture (*p* Value < 0.001 *)				
	1	2	3	4	5	1	2	3	4	5	1	2	3	4	5
1	0	0	0	0	0	0	0	1	0	1	0	0	2	1	0
2	0	0	0	0	0	1	2	1	2	0	0	0	2	2	1
3	0	3	6	1	1	0	1	0	2	3	0	0	0	7	2
4	0	2	4	2	1	0	0	1	7	0	0	2	0	1	0
5	0	0	2	1	0	0	1	0	0	0	0	0	1	1	1

* From ordinal paired test using clmm function from R ordinal package.

**Table 5:** Endocannabinoid compounds and related compounds analysis.

N = 24	Global P	Lineal P	Baseline versus 30 min (*p* Value)	Baseline versus 60 min (*p* Value)	Baseline versus 120 min (*p* Value)
og (ng/mL)	0.254	0.418	0.458	0.425	0.284
aea (ng/mL)	0.556	0.968	0.420	0.670	0.409
dglea (ng/mL)	0.174	0.940	0.261	0.154	0.553
dhea (ng/mL)	0.451	0.955	0.701	0.381	0.610
lea (ng/mL)	0.536	0.869	0.244	0.549	0.454
oea (ng/mL)	0.245	0.944	0.730	0.478	0.230
poea (ng/mL)	0.001	0.649	0.867	0.846	0.002
pea (ng/mL)	0.712	0.866	0.476	0.802	0.974
ag (ng/mL)	0.400	0.455	0.284	0.164	0.581
lg (ng/mL)	0.624	0.944	0.409	0.489	0.530
ratio_oea_aea	0.261	0.429	0.301	0.496	0.664
ratio_oea_pea	<0.001	0.572	0.443	0.636	0.654
ratio_pea_aea	0.844	0.913	0.880	0.634	0.948

og, 2-oleoyl glycerol; aea, N-arachidonoyl ethanolamine (anandamide); dglea, N-di-homo-y-linolenoyl ethanolamine; dhea, N-docosahexaenoyl ethanolamine; lea, N-linolenoyl ethanolamine; oea, N-oleoyl ethanolamine; poea, N-palmitoleoyl ethanolamine; pea, N-palmitoyl-ethanolamine; ag, 2-arachidonoyl glycerol; lg, 2-linoleoyl glycerol.

**Table 6:** Endocannabinoid compounds and related compounds analysis in women.

Women N = 12	Global P	Lineal P	Baseline versus 30 min (*p* Value)	Baseline versus 60 min (*p* Value)	Baseline versus 120 min (*p* Value)
og (ng/mL)	0.107	0.346	0.549	0.606	0.162
aea (ng/mL)	0.300	0.906	0.414	0.960	0.380
dglea (ng/mL)	0.017	0.564	0.316	0.019	0.792
dhea (ng/mL)	0.311	0.704	0.871	0.573	0.395
lea (ng/mL)	0.308	0.984	0.218	0.996	0.329
oea (ng/mL)	0.040	0.943	0.707	0.248	0.127
poea (ng/mL)	0.001	0.627	0.690	0.997	0.004
pea (ng/mL)	0.253	0.948	0.667	0.864	0.579
ag (ng/mL)	0.963	0.638	0.415	0.512	0.891
lg (ng/mL)	0.458	0.656	0.351	0.504	0.784
ratio_oea_aea	0.208	0.265	0.332	0.612	0.891
ratio_oea_pea	0.010	0.564	0.558	0.684	0.735
ratio_pea_aea	0.930	0.7	0.564	0.538	0.668

og, 2-oleoyl glycerol; aea, N-arachidonoyl ethanolamine (anandamide); dglea, N-di-homo-y-linolenoyl ethanolamine; dhea, N-docosahexaenoyl ethanolamine; lea, N-linolenoyl ethanolamine; oea, N-oleoyl ethanolamine; poea, N-palmitoleoyl ethanolamine; pea, N-palmitoyl-ethanolamine; ag, 2-arachidonoyl glycerol; lg, 2-linoleoyl glycerol.

## Data Availability

The generation and analysis of the data sets within this study are not projected to be open to access beyond the core research group. This is because the participants’ consent forms and ethical approval did not include provisions for public accessibility. However, we follow a controlled data-sharing collaboration model, as the informed consent documents signed by the participants allow for regulated collaboration with other researchers for study-related research. The data described in the manuscript, alongside the codebook and analytic code, will be available upon request. Researchers interested in this study can reach out to the corresponding author, Olga Castañer (ocastaner@researchmar.net).

## Supplementary Materials

The following supporting information can be downloaded at: https://www.mdpi.com/article/10.3390/nu16183180/s1, Table S1: Consort checklist.
